# Advancing Evidence Generation for Circulating Tumor DNA: Lessons Learned from A Multi-Assay Study of Baseline Circulating Tumor DNA Levels across Cancer Types and Stages

**DOI:** 10.3390/diagnostics14090912

**Published:** 2024-04-27

**Authors:** Brittany A. McKelvey, Hillary S. Andrews, Frederick L. Baehner, James Chen, Carin R. Espenschied, David Fabrizio, Vanessa Gorton, Claire Gould, Justin Guinney, Greg Jones, Xiangyang Lv, Michael S. Nahorski, Melanie R. Palomares, Gary A. Pestano, Mark Sausen, Alain Silk, Nicole Zhang, Zhihong Zhang, Mark D. Stewart, Jeff D. Allen

**Affiliations:** 1 Friends of Cancer Research, Washington, DC 20036, USA; 2 Exact Sciences Corp., Madison, WI 53719, USA; 3 Tempus AI, Inc., Chicago, IL 60654, USA; 4 Guardant Health, Inc., Redwood City, CA 94063, USA; 5 Foundation Medicine, Inc., Cambridge, MA 02141, USA; 6 Predicine, Hayward, CA 94545, USA; 7 Biodesix Inc., Louisville, CO 80027, USA; 8 NeoGenomics Laboratories, Fort Myers, FL 33912, USA; 9 Burning Rock, Irvine, CA 92617, USA; 10 Personal Genome Diagnostics, Labcorp, Baltimore, MD 21224, USA

**Keywords:** ctDNA, cancer, biomarker

## Abstract

Circulating tumor DNA (ctDNA) holds promise as a biomarker for predicting clinical responses to therapy in solid tumors, and multiple ctDNA assays are in development. However, the heterogeneity in ctDNA levels prior to treatment (baseline) across different cancer types and stages and across ctDNA assays has not been widely studied. Friends of Cancer Research formed a collaboration across multiple commercial ctDNA assay developers to assess baseline ctDNA levels across five cancer types in early- and late-stage disease. This retrospective study included eight commercial ctDNA assay developers providing summary-level de-identified data for patients with non-small cell lung cancer (NSCLC), bladder, breast, prostate, and head and neck squamous cell carcinoma following a common analysis protocol. Baseline ctDNA levels across late-stage cancer types were similarly detected, highlighting the potential use of ctDNA as a biomarker in these cancer types. Variability was observed in ctDNA levels across assays in early-stage NSCLC, indicative of the contribution of assay analytical performance and methodology on variability. We identified key data elements, including assay characteristics and clinicopathological metadata, that need to be standardized for future meta-analyses across multiple assays. This work facilitates evidence generation opportunities to support the use of ctDNA as a biomarker for clinical response.

## 1. Introduction

The measurement of circulating tumor DNA (ctDNA) has emerged as a promising surrogate for disease burden and, by extension, a research tool to rapidly evaluate clinical response across a myriad of therapeutic interventions. Emerging data continue to build momentum around the various clinical and regulatory applications of ctDNA in oncology, including predicting a patient’s response to therapy [1,2,3,4,5]. The use of ctDNA to predict clinical response could enable faster identification and development of more effective drugs and, importantly, support regulatory decision-making as an early endpoint predicting long-term clinical outcomes [6,7,8,9]. Early endpoints that are “reasonably likely to predict a clinical benefit” are increasingly important in oncology drug development to shorten development timelines and get effective drugs to patients faster [10]. The U.S. Food and Drug Administration’s (FDA) Draft Guidance on the Use of Circulating Tumor DNA for Early-Stage Solid Tumor Drug Development highlights the use of ctDNA as an early endpoint in clinical trials; however, it also states that further data are needed to support its use [11].

Although advancements in technologies are leading to more sensitive and precise tools for detecting and measuring ctDNA, all technologies have inherent limitations and variability [12]. Further, ctDNA may not be detected at sufficient levels to allow informative analysis across all cancer types and stages. Thus, it is important to understand the extent to which heterogeneity in ctDNA levels across different cancer types and stages stems from tumor-specific factors, such as tumor shed rates, and technical factors, such as the dynamic range of the assay for interpreting ctDNA measurement. Several efforts have assessed the landscape of ctDNA detection across cancer types in large real-world evidence cohorts [13,14,15]. However, these data are specific to a single technology, laboratory, or assay and are focused largely in the advanced or metastatic setting where tumor biology may be fundamentally different from earlier-stage cancer in which the application of ctDNA as an early endpoint may be especially valuable. To evaluate the technical and biological variability across cancer types and assays, a multi-assay study was conducted to investigate baseline ctDNA levels (ctDNA levels prior to current cancer treatment) in multiple cancer types and stages. We generated descriptive statistics to compare trends in baseline ctDNA levels across assays by cancer type and stage through a collaborative effort with multiple commercial assay developers. While informative, our findings identified key considerations required to support broad data harmonization efforts to generate evidence for the use of ctDNA as an early endpoint across assays and clinical settings.

## 2. Materials and Methods

Each assay developer retrospectively aggregated data from their database following a common data analysis protocol, which specified data elements and analyses to generate summary-level statistics across five cancer types (see Supplementary Materials, Tables S1–S3), with each assay dataset defined as a cohort. Patients included in this analysis were adult patients, aged 18 or older at the date of ctDNA sample collection, diagnosed with cancer, and had either not yet initiated anti-cancer therapy or had not received anti-cancer therapy at the time of baseline sampling (see Supplementary Materials, Section S3). Non-small cell lung cancer (NSCLC), bladder, breast, prostate, and head and neck squamous cell carcinoma (HNSCC) cancers were analyzed due to the availability of baseline ctDNA data from at least two assay developers. Patients were included if they had known early- or late-stage cancer at the time of baseline sampling. Summary-level clinical and demographic characteristics were reported for each cohort if known.

The pre-analytic cell free DNA (cfDNA) minimal technical data elements (MTDEs) [16] proposed by the Blood Profiling Atlas in Cancer (BloodPAC) Consortium were used to ensure that pre-analytical variability was similarly controlled across cohorts to reduce the impact of pre-analytical factors. Assay characteristics were reported and aggregated across developers. No patient-level identifiers and, thus, no protected health information were revealed or exchanged in this process.

Summary-level data on baseline ctDNA levels for specific cancer types and stages were reported by cohort. Following the ctDNA to Monitor Treatment Response (ctMoniTR) project [9], summary-level statistics of sample size, median, mean, standard deviation (SD), Interquartile Range (IQR), minimum and maximum for each of the median variant allele frequency (VAF), maximum VAF, and mean VAF were reported for baseline ctDNA levels. Descriptive statistics were used.

## 3. Results

### 3.1. Assay Characteristics

Eight commercial assays measuring baseline ctDNA were blinded and included in the analysis (labeled Cohort A-I). Five assays (62.5%) were tumor-informed (i.e., mutations identified in the primary tumor tissue that are tracked in the plasma), and three (37.5%) were tumor-naïve (i.e., mutations were detected de novo from the plasma). All but one assay (87.5%) used next-generation sequencing (NGS); the remaining assay used droplet digital PCR (ddPCR). Half (4/8) of the assays did not conduct clonal hematopoiesis of indeterminate potential (CHIP) filtering, three (37.5%) used bioinformatic methods, and one (12.5%) used germline sequencing methods to filter for CHIP variants. All assays assessed single nucleotide variants (SNVs) with a median limit of detection (LOD) of 0.2% VAF (range, 0.0011–0.5%).

### 3.2. Sample Characteristics

Across the eight cohorts, data from early- and late-stage samples were provided for NSCLC, with 2357 early-stage and 62,994 late-stage samples and 87,209 total samples across all five late-stage cancer types (Table 1). Most cohorts did not have data available for AJCC staging, prior anti-cancer treatments, recurrence or progression status, and the type of recurrence. The timing of ctDNA sampling relative to diagnosis varied across cohorts, with long durations observed in late-stage cancers.

### 3.3. Baseline ctDNA Levels

**Figure 1:**
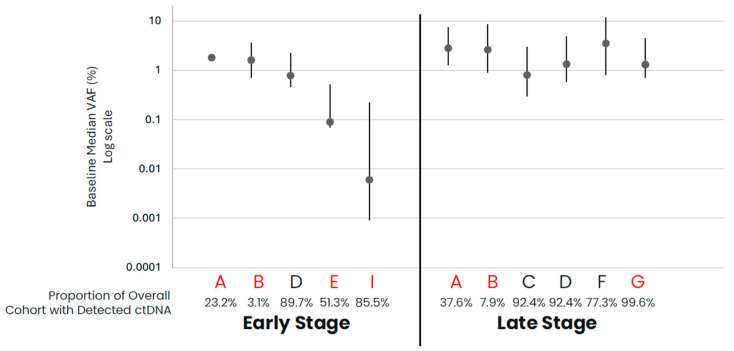
NSCLC baseline ctDNA levels for samples with detected ctDNA. Median VAF (IQR) ctDNA levels for samples with detected ctDNA by cohort, with the proportion of total cohort samples with detected ctDNA shown below the graph. Cohorts in red are tumor-informed assays, and cohorts in black are tumor-naïve assays. Median VAF—the median of VAF values from all somatic tumor-derived variants.

**Figure 2:**
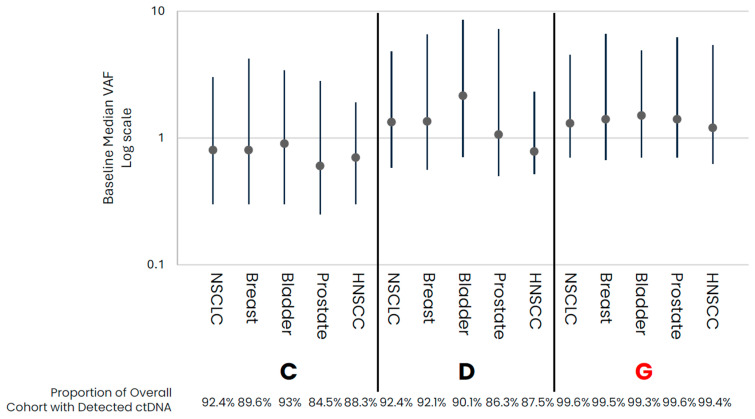
Late-Stage baseline ctDNA levels for samples with detected ctDNA. Median (IQR) VAF ctDNA levels for samples with detected ctDNA by cohort, with the proportion of total cohort samples with detected ctDNA shown below the graph. Colored points highlight the different cancer types. Cohorts in red are tumor-informed assays, and cohorts in black are tumor-naïve assays. Median VAF—the median of VAF values from all somatic tumor-derived variants.

In comparing early- versus late-stage NSCLC, the frequency of ctDNA detection varied across cohorts, with late-stage NSCLC having a higher proportion of samples with detected ctDNA than early-stage in data from assays that had both early- and late-stage data available (Table 1, Figure 1). For those samples with detected ctDNA, late-stage NSCLC samples generally appeared to have higher levels as compared to early-stage samples, with cohort variability observed. Across the late-stage cancer types evaluated, baseline ctDNA was similarly detected across most samples across cohorts (Table 1, Figure 2). For the three assays with data available across all five late-stage cancer types, baseline ctDNA levels were similar across cancer types and assays.

## 4. Discussion

This collaborative effort evaluated baseline ctDNA levels by cancer type and stage across different assays to identify overall trends and considerations to support future data harmonization efforts to generate evidence for the use of ctDNA as an early endpoint. Overall, baseline ctDNA levels across late-stage NSCLC, breast, bladder, prostate, and HNSCC cancers were similarly detected, suggesting the potential opportunity to use ctDNA as a clinical biomarker in these cancer types. Conversely, more variability in ctDNA levels across assays was observed in early-stage NSCLC than in late-stage disease, highlighting the critical need to consider factors such as assay analytical performance and methodology for evaluating ctDNA in this setting [17].

Assay characteristics, including the intended use, features assessed, and analytical performance, were variable, leading to difficulties in interpreting aggregated data. The development of common data standards could help allow more robust comparisons across assay datasets [18]. The heterogeneity in approaches to identifying SNVs (e.g., tumor-informed or naïve) and CHIP filtering can cause variability between assays for samples determined to have detected ctDNA. For example, our study explored mean, median, and maximum VAF (median reported herein) and observed biases in mean and maximum VAF values in some cohorts due to conflation by high VAF values derived from suspected germline variants. However, median VAF may also misrepresent data when ctDNA levels are low (e.g., in the stochastic range) and bias against the lower range of detection. Therefore, setting standards for how ctDNA levels are reported across assays as well as a clear understanding of the methodology for obtaining ctDNA values are critical.

Real-world data are a valuable source of data for analyses but provide challenges in meta-analyses due to data missingness and heterogeneity [19]. The availability of clinicopathological data was generally lacking across cohorts in this study. Each developer could confidently categorize their samples as either early- or late-stage disease. Many could not provide the AJCC clinical staging, which may impact observed ctDNA levels given differences in tumor shedding by stage, and data on prior anti-cancer treatments and recurrence or progression status were mostly unknown. The lack of available clinical data was not surprising given that assay developers included in this analysis were clinical laboratories providing testing as a service to health systems and may not have routine access to comprehensive clinical data for each sample tested. However, an understanding of prior treatment is critical to define baselines, as samples may be included from patients who are treatment-naïve, as well as patients who have received prior anti-cancer treatment and subsequently recurred or progressed. Due to unknown clinicopathological factors, treatment or surgical intervention status, and sample collection timing from diagnosis, significant cohort heterogeneity may complicate comparisons across cohorts.

The timing from diagnosis to sampling was heterogeneous, especially in late-stage cancers, which could be affected by the intended use of the test when ctDNA analysis is conducted during the patient journey. This variability, along with other anti-cancer treatments or modalities that could impact ctDNA levels, highlights the importance of defining minimal criteria for the length of time between diagnosis and sampling. This may potentially avoid variability surrounding long timeframes. As a result, it is important to identify and standardize key data elements, including assay characteristics and clinicopathological data, to facilitate robust evidence generation to support the use of ctDNA as an early endpoint, leading to more harmonized and effective use of ctDNA in future clinical research and care.

## 5. Conclusions

To support the future use of ctDNA as an early endpoint, meta-analyses across assays, supported by appropriate clinicopathological metadata, are needed for multiple cancer types and stages. This collaborative effort has enabled the evaluation of baseline ctDNA levels by cancer type and stage across different assays to identify overall trends and considerations. This effort supports future data harmonization efforts to validate the use of ctDNA as an early endpoint, highlighting the potential opportunity to use ctDNA as a clinical biomarker in late-stage NSCLC, breast, bladder, prostate, and HNSCC cancers due to the similar detection of baseline ctDNA levels across these cancer types. However, more variability in ctDNA levels across assays was observed in early-stage NSCLC than in late-stage types, underscoring the importance of evaluating factors such as assay analytical performance and methodology in this setting.

Given the heterogeneity of data from real-world sources, routine collection and analysis of ctDNA from patients in oncology clinical trials may provide more comprehensive and standardized clinical data and assure within-cohort control over technical variability. The development of common data standards and an understanding of assay technological features and key performance characteristics can improve the poolability of data generated using different assays. The learnings from this study, such as the need to address the heterogeneity in approaches to identifying SNVs and the challenges posed by assay characteristic variability, underscore the complexity of interpreting aggregated data and the importance of developing methodological approaches to combine data from different trials and assays. These highlighted data needs can facilitate future pooled analyses to generate robust evidence to support the use of ctDNA as a biomarker and early endpoint, setting the stage for a more harmonized and effective approach to oncology drug development and patient care.

## Figures and Tables

**Table 1:** Clinicopathological characteristics and baseline ctDNA VAFs by assay cohort ^1^.

**A: Early- and Late-Stage Non-Small Cell Lung Cancer (NSCLC)**												
	**NSCLC**											
	**Early Stage**					**Late Stage**						
**Cohort**	**A**	**B**	**D**	**E**	**I**	**A**	**B**	**C**	**D**	**F**		**G**
**N** (samples)	245	1873	679	78	131	1232	31,889	23,157	2452	264		4000
**Age** (median, years)	70	70	70	Unkn	63	69	67	68	Unkn	70		73
**Gender** (% female)	48	49	49	49	35	49	50	52	49	52		53
**Clinical Stage**												
I	5	19	15	53	48	0	0	0	0	0		0
II	2	15	17	28	17	0	0	0	0	0		0
III	11	29	68	19	35	5	1	0	0	15		0
IV	0	0	0	0	0	13	7	0	100	82		0
Unknown	82	37	0	0	0	82	92	100	0	3		100
**Prior Anti-Cancer Treatments**												
Known Tx	18	21	0	0	0	18	3	0	0	0		0
None	1	42	0	0	100	11	6	0	0	0		0
Unknown	81	37	100	100	0	71	91	100	100	100		100
**Recurrence/Progression Status**												
No prior cancer	2	11	0	0	100	0	1	0	0	0		0
Unknown	95	86	100	100	0	97	98	100	100	100		100
**Timing of Sampling**, days from diagnosis to sampling, (median (IQR))	461 (145.5, 891.5)	40 (16, 134.3)	22 (13, 36)	<84	1 (1, 1)	602.5 (334, 850.8)	29 (13, 31.8)	18 (7, 52)	17 (9, 36)	<84		Unkn
**Frequency of ctDNA Detected in Samples** (%)	23.2	3.1	89.7	51.3	85.5	37.6	7.9	92.4	92.4	77.3		99.6
**Median VAF** (IQR)	1.8 (0.7, 2.3)	1.6 (0.9, 2)	0.78 (0.32, 1.44)	0.09 (0.02, 0.42)	0.001 (0.001, 0.2)	2.8 (1.5, 4.6)	2.6 (1.7, 5.8)	0.8 (0.5, 2.2)	1.33 (0.75, 3.47)	3.49 (2.67, 8.21)		1.3 (0.6, 3.2)
**B: Late-Stage Breast, Bladder, Prostate, and HNSCC Cancers**												
	**Late Stage**											
	**Breast**			**Bladder**			**Prostate**			**HNSCC**		
**Cohort**	**C**	**D**	**G**	**C**	**D**	**G**	**C**	**D**	**G**	**C**	**D**	**G**
**N** (samples)	2572	1020	6940	500	282	577	1100	633	9502	274	136	546
**Age** (median, years)	62	61	64	72	71	73	70	68	74	64	62	64
**Gender** (% female)	98	100	99	29	26	25	0	0	0	22	24	23
**Clinical Stage**												
I	0	0	0	0	0	0	0	0	0	0	0	0
II	0	0	0	0	0	0	0	0	0	0	0	0
III	0	0	0	0	0	0	0	0	0	0	0	0
IV	0	100	0	0	100	0	0	100	0	0	100	0
Unknown	100	0	100	100	0	100	100	0	100	100	0	100
**Prior Anti-Cancer Treatments**												
Known Tx	0	0	0	0	0	0	0	0	0	0	0	0
None	0	0	0	0	0	0	0	0	0	0	0	0
Unknown	100	100	100	100	100	100	100	100	100	100	100	100
**Recurrence/Progression Status**												
No prior cancer	0	0	0	0	0	0	0	0	0	0	0	0
Unknown	100	100	100	100	100	100	100	100	100	100	100	100
**Timing of Sampling**, days from diagnosis to Ssmpling, (median (IQR))	262 (16, 1220)	35 (20, 75)	Unkn	70 (11, 557)	241 (35, 741)	Unkn	126.5 (9, 1130.5)	42 (21, 1255)	Unkn	97.5 (9, 728.5)	34 (24, 135)	Unkn
**Frequency of ctDNA Detected in Samples** (%)	89.6	92.1	99.5	93	90.1	99.3	84.5	86.3	99.6	88.3	87.5	99.4
**Median VAF** (IQR)	0.8 (0.5, 3.4)	1.35 (0.79, 5.19)	1.4 (0.73, 5.22)	0.9 (0.6, 2.5)	2.15 (1.44, 6.36)	1.5 (0.8, 3.4)	0.6 (0.35, 2.2)	1.06 (0.56, 6.14)	1.4 (0.7, 4.8)	0.7 (0.4, 1.2)	0.78 (0.26, 1.53)	1.2 (0.58, 4.19)

^1^ Tx: treatment, Unkn: unknown, IQR: Interquartile Range. Cohorts in red are tumor-informed assays, and cohorts in black are tumor-naïve assays. Median VAF—the median of VAF values from all somatic tumor-derived variants.

## Data Availability

The patient-level datasets presented in this article are not readily available due to patient privacy and legal restrictions.

## Supplementary Materials

The following supporting information can be downloaded at https://www.mdpi.com/article/10.3390/diagnostics14090912/s1: Supplementary Protocol: Introduction, Objective, Study Cohort, Data Collection, Statistical Considerations, Data Dictionary and Table S1: Study variable definitions; Table S2: Pre-analytic technical specification elements; Table S3: ctDNA results.
