# Supplementary material for: Advancing Evidence Generation for Circulating Tumor DNA: Lessons Learned from A Multi-Assay Study of Baseline Circulating Tumor DNA Levels across Cancer Types and Stages
Source: Diagnostics (Basel). 2024 Apr 27;14(9):912. doi: 10.3390/diagnostics14090912 (PMC11083008; doi:10.3390/diagnostics14090912)
Supplement: Supplementary file 1 [file diagnostics-14-00912-s001.zip › diagnostics-2886304-supplementary.pdf]

Survey of Baseline ctDNA Levels
Statistical Analysis Plan

Establishing evidence regarding baseline sensitivity metrics for ctDNA detection across cancer types, stages, and assays through a collaborative effort involving multiple diagnostic developers

Table of Contents

Introduction 1
Objective 1
Study Cohort 2
- Inclusion Exclusion Criteria 2

Data Collection 2
- Clinical and Demographic Characteristics 2
- Pre-Analytical Specification 3
- ctDNA Assay Characteristics 3
- ctDNA Results 3

Statistical Considerations 3
- Descriptive Analysis of Study Characteristics 3
- Descriptive Analysis of Assay Characteristics 4
- Distribution of Baseline ctDNA Levels 4

Data Dictionary 5

1. Introduction

The ability to use ctDNA as an early endpoint in oncology clinical trials may vary by tumor type and by stage. The biology of different tumor types at different stages may impact tumor shedding of ctDNA and the availability of a sufficient dynamic range of measurable ctDNA to allow for meaningful use as an endpoint. Further, ctDNA assessments can vary by assay and technology used to detect ctDNA, which may yield variable results that are difficult to interpret across studies using different technologies. There is not extensive data describing the extent and range of detectable baseline levels of ctDNA across different cancer types as measured by different technologies, as well as how ctDNA levels vary between early and late stages in different cancer types. A landscape assessment of baseline ctDNA levels could help support future meta-analyses and use of ctDNA as an endpoint in additional tumor types and/or stages.

2. Objectives:

Measure the Variability in ctDNA Levels by Cancer Type and Stage

Perform a descriptive analysis to compare trends in baseline ctDNA levels (ctDNA levels prior to a current cancer treatment) between cancer types and stages.

Sub-Objectives:

- Compare baseline ctDNA level trends across cancer types in late-stage disease
- Compare baseline ctDNA level trends across assays within the same cancer type and disease setting
- Compare overall trends of baseline ctDNA levels across stages within the same cancer type

Approach: Summary-level data on baseline ctDNA levels for specific cancer types will be reported by assay developers. Subgroup analyses will be conducted to account for variability in sample and assay characteristics.

3. Study Cohort:

3.1 Cohort Inclusion and Exclusion Criteria

Inclusion Criteria:

- Adult patient, age 18 or older at date of ctDNA sample collection.
- Patient is diagnosed with cancer of the following types. Cancer types were chosen based on the availability of baseline ctDNA data across participating assay developers. Cancer types in which at least two assay developers had relevant datasets to contribute are included.
    - Bladder Cancer
    - Breast Cancer
    - Head and Neck Squamous Cell Carcinoma (HNSCC)
    - Non-Small Cell Lung Cancer (NSCLC)
    - Prostate Cancer
- Known stage of cancer at time of sampling.
    - Known AJCC clinical, anatomical staging preferred (stage I-IV).
        - Staging should not take into account molecular alterations
    - At minimum, known “early” or “late” stage (i.e., stage category).
    - Clinical staging will be categorized into either (see Table 1 for exceptions to definitions):
    - Early-stage: Localized cancer amenable to intervention with curative intent
    - Late-stage: Cancer that has metastasized and is not amenable to therapy with curative intent
- Patient, at time of baseline sampling, has not yet initiated anti-cancer therapy
    - Note: Patient may have received prior anti-cancer therapy for prior cancer (or unknown treatment history) but must not be actively undergoing treatment at time of sampling.

Exclusion Criteria:

- Patients receiving anti-cancer therapy at the time of sampling

4. Data Collection:

4.1 Clinical and Demographic Characteristics

The following clinical and demographic characteristics will be collected for the study cohort. See data dictionary Table 1 for additional granularity on data elements.

- Age
- Sex
- Tumor Type
- Tumor Subtype, *if known*
- AJCC Clinical Stage, at time of sampling*, if known*
- Stage Category, at time of sampling (i.e., early or late stage, as defined above)
- Anti-Cancer Treatments Received Prior to Sampling, *if known*
- Recurrence or Progression Status, *if known*
- Type of Recurrence, *if known*
- Quarter, Year Sample Collected

- Timing of Sampling Relative to Current Diagnosis

4.2 Pre-Analytical Specification

To ensure that pre-analytical variability is minimal and therefore similar quality of ctDNA across datasets, we adopt the plasma collection methods noted in data dictionary Table 2. These recommendations are based on the ctMoniTR project, and the pre-analytic cfDNA minimal technical data elements (MTDEs) proposed by the Blood Profiling Atlas in Cancer (BloodPAC) Consortium. Assay developers will note their alignment with each method category.

4.3 ctDNA Assay Characteristics

The following assay characteristics will be collected for the ctDNA assays. See data dictionary Table 1 for additional granularity on ctDNA assay characteristics noted below.

- Assay type
- Intended use
- Sequencing or other platform
- Molecular concentration metric
- CHIP filtering method, if applicable
- Minimal 95% LOD, composite (across alterations)
    - SNV/indel LOD
- Biomarker features assessed

4.4 ctDNA Results

The following summary-level data of ctDNA detection will be collected from each assay developer by cancer type and stage (see data dictionary Table 3 for further granularity).

- Number of patients
- Number of baseline samples
- Molecular concentration
- Volume of plasma
- Mean VAF
- Median VAF
- Max VAF
- Frequency of ctDNA detection

5. Statistical Considerations

5.1 Descriptive Analysis of Patient Characteristics

Clinical and demographic characteristics of each dataset by tumor type and stage will be summarized using descriptive statistics. Categorical variables will be reported as total counts and percentages, while continuous variables will be reported as mean, standard deviation, median, interquartile range (IQR), and range to determine appropriate categories. Given the range of data for the continuous variables of age and timing of sampling, categories will be fitted to allow for these data elements to be categorical as well.
Categorical data elements to display:

- Age
- Sex
- Tumor Type
- Stage Category

- Quarter, Year Sample Collected
- Timing of Sampling Relative to Current Diagnosis
- Tumor Subtype*, if known*
- Clinical Stage, *if known*
- Anti-Cancer Treatments Received Prior to Sampling, *if known*
- Recurrence or Progression Status, *if known*
- Recurrence Type*, if known*

5.2 Descriptive Analysis of Assay Characteristics

Statistical analyses for the assay characteristics included in the study will be descriptive to understand the variability in ctDNA assessment across data sources. Results will be summarized for the percent of assays in the study with the following categorical variables:

- Assay type
- Intended use
- Sequencing or other platform
- Molecular concentration metric
- CHIP filtering method
- Biomarker features assessed

The median and interquartile (IQR) will be reported for the minimal 95% LOD of the assays included in the analysis for each alteration type, as applicable to the individual assay (i.e., composite, SNV/indel, fusion, CNV).

5.3 Distribution of Baseline ctDNA Level Analyses

5.3.1 Distribution of Baseline ctDNA Levels

The summary-level statistics will be provided from each individual data source and the distribution of ctDNA levels will be compared across assays, cancer types, and stage category. Summary-level statistics for the plasma volume and molecular concentration of input into the assay will be reported.

5.3.1.1 Distribution of Baseline ctDNA Levels in Late-Stage Disease

Quantitative

The distribution of baseline ctDNA levels in late-stage disease will be compared across different cancer types. Each developer will report the summary-level statistics of sample size, median, mean (SD), IQR, min, and max for each of the Median VAF, Max VAF, and Mean VAF for each late-stage cancer type. An adaption of box and whisker facet grid plots will be used to visually demonstrate the distribution of baseline ctDNA levels, and will be created for the Median VAF, Max VAF, and Mean VAF. Each line will demonstrate the distribution of ctDNA levels in a different late-stage cancer type within a single assay dataset.

Tables noting the median, mean (SD), IQR, min, and max for each of the Median VAF, Max VAF, Mean VAF, and MTM/mL for each late-stage cancer type within each assay will also display the data. Each assay will be categorized by assay type (tumor-informed or tumor-naïve/liquid only) to allow comparison by assay type.

Qualitative

In a descriptive exploratory analysis, the percentage of baseline samples in which ctDNA is detected within a cancer type per dataset will be calculated. Trends will be compared across datasets and cancer types.

5.3.1.2 Distribution of Baseline ctDNA Levels in Early-Stage Disease
The above analyses in 5.3.1.1 will be conducted for early-stage cancer types.

6. Data Dictionary

Data dictionary is provided below for reference.

Supplemental Table 1. Study variable definitions.

Category	Variable Name	Definition
**Clinical and Demographic Characteristics**	**Year sample collected**	• The calendar year in which the ctDNA sampling occurred, as well as quarter (Q1, 2, 3, 4) (categorical)
	**Year sample collected**	• The calendar year in which the ctDNA sampling occurred (categorical)
	**Age at index**	• Age at the time of ctDNA sampling (continuous)
	**Age at index (categorical)**	• Age at the time of ctDNA sampling (categorical)
	**Sex**	• Male<br>• Female<br>• Other / Unknown
	**Tumor Type**	• The histological classification of cancer type. Included cancer types:<br>o Bladder Cancer<br>o Breast Cancer<br>o Head and Neck Squamous Cell Carcinoma (HNSCC)<br>o Non-Small Cell Lung Cancer<br>o Prostate Cancer
	**Tumor Subtype**	• The histological subtype classification, if known. Categories for each cancer type chosen:<br>o Bladder Cancer<br>• Urothelial<br>• Squamous Cell<br>• Adenocarcinoma<br>• Small Cell<br>• Sarcoma<br>• Unknown<br>o Breast Cancer (Pathological)<br>• Ductal Carcinoma In Situ<br>• Lobular Carcinoma In Situ<br>• Invasive Ductal<br>• Invasive Lobular<br>• Unknown<br>o Breast Cancer (Hormone)<br>• HR positive (ER/PR)<br>• Triple Negative<br>• HER2+<br>o Head and Neck Squamous Cell Carcinoma (HNSCC)<br>o Non-Small Cell Lung Cancer<br>• Squamous Cell<br>• Adenocarcinoma<br>• Large Cell

		• Unknown<br>o Prostate Cancer<br>• Adenocarcinoma<br>• Small Cell<br>• Squamous Cell<br>• Sarcoma<br>• Neuroendocrine<br>• Unknown
	**Clinical Stage**	• AJCC clinical stage of disease at the time of ctDNA sampling, if known (categorical)<br>o I<br>o II<br>o III<br>o IV<br>o Unknown
	**Stage Category**	• Dichotomous staging categorization at the time of ctDNA sampling (categorical)<br>o Early: localized cancer amenable to local intervention with curative intent<br>o Late: cancer that has metastasized and is not amenable to local intervention<br>o Exceptions: HNSCC: Stage IVA and IVB will be considered early-stage if the disease is locally advanced and the patient is undergoing curative intent therapy
	**Prior anti-cancer treatments**	• Known anti-cancer treatments received prior to the time of ctDNA sampling that may impact the biological understanding of baseline ctDNA levels<br>o Prior surgical intervention<br>o Prior radiation therapy<br>o Prior systemic therapy<br>o Prior other treatment<br>o Prior multiple treatments (treatment belonging to multiple categories)<br>o None<br>o Not documented/Unknown
	**Recurrence or Progression Status**	• Any prior cancer diagnosis, if known<br>o No prior cancer diagnosis<br>o Recurrence or relapse after previous treatment for current cancer<br>o Unknown
	**Type of Recurrence**	• Type of recurrence, if known<br>o No prior cancer diagnosis<br>o Locoregional recurrence<br>o Distant recurrence<br>o Unknown type of recurrence
	**Timing of sampling**	• Given the variability in timing of baseline sampling across datasets, the time (in days) between diagnosis and sample collection will be recorded and reported through summary statistics (median, IQR).<br>• This will inform categorical variables for timing of sampling.
**ctDNA Assay Characteristics**	**Assay type**	• The approach to assessing ctDNA, whether informed or uninformed by the primary tumor<br>o Tumor-informed<br>o Tumor-naïve (liquid only)
	**Intended use**	• The current intended use of the assay:

		o Early-stage only<br>o Late-stage only<br>o All stages
	**Sequencing or other platform**	• The platform to determine ctDNA detection<br>o Next-generation sequencing (NGS)<br>o Droplet digital PCR (ddPCR)
	**Molecular Concentration Metric**	• The metric used to report the total amount of non-encapsulated cell-free DNA isolated from the plasma and used for the assay<br>o ng of DNA<br>o Copies/mL<br>o Other (define)
	**CHIP filtering method**	• If applicable, the method for filtering clonal hematopoiesis of indeterminate potential (CHIP)<br>o Bioinformatic only<br>o Germline sequencing<br>o Buffy coat sequencing<br>o Other (define)<br>o None
	**Minimal LOD**	• The 95% limit of detection (LOD) for the assay used to determine ctDNA detection. The lowest concentration at which 95% of positive samples are detected. Reported by % allele frequency (continuous)<br>• Composite LOD across all alterations assessed<br>• Separate LODs will be reported for each alteration type:<br>o SNVs/Indels<br>o Fusions<br>o Copy number variants<br>• A short description will be provided of how the LOD was derived
	**Biomarker features assessed**	• The types of biomarker features assessed by the assay<br>o Somatic nucleotide variants (SNV)<br>o Structural variant alterations<br>• Indels<br>• Fusions<br>• Copy number variants<br>o Methylation<br>o Fragmentation

Supplemental Table 2. Pre-Analytic Technical Specification Elements

Element	Proposed Method
Timepoints	Baseline: Pre-treatment collection
Tube types	Blood collection tube validated for use with the assay, specify specific tube brand
Material collected	Plasma from whole blood
Blood fractionation method	Centrifugation
Number of centrifugation spins	Double spin
Time to fractionation	Within validated specifications for blood collection tube used
Holding temperature	4°C for isolated DNA<br>Room temperature for spun plasma in blood tubes
Storage temperature	-20°C if short term storage<br>-80°C if long term storage

DNA quantification	Fluorometric method (e.g. Qubit, nanodrop)

Collection parameters fall in line with the Blood Profiling Atlas in Cancer (BloodPAC) Consortium Pre-Analytic cfDNA Minimal Technical Data Elements (MDTEs)

Supplemental Table 3. ctDNA Results

Variable Name	Definition
**Number of Patients**	• The number of patients included in the dataset, per cancer type and stage category, including patients with non-detectable samples
**Number of Samples**	• The number of samples included in the dataset, per cancer type and stage category, including non-detectable samples
**Molecular Concentration**	• The median and IQR of the total amount of non-encapsulated cell-free DNA isolated from the plasma and used for the assay for all samples per cancer type and stage category, in ng of DNA<br>• Conversion of copies/mL to ng of DNA
**Volume of Plasma Extracted**	• The median and IQR of the total volume of plasma collected from each blood samples, in mL, for all samples per cancer type and stage category
**Variant Allele Frequency**	• The number of mutant alleles divided by the total number of mutant and wildtype alleles, per variant/indel<br>• All somatic tumor-derived variants included, regardless of the gene, mutation, or type of mutation (missense, nonsense, silent, frameshift) as well as indels<br>• Each developer will be responsible for removing variants that fail to meet their own bioinformatics filters, ensuring that only tumor-derived alterations are included. This includes removal of low-quality or spurious variants that fall below an assay's established LOD, variants that may be sequencing errors/artifacts, or variants with insufficient sequencing coverage
**Mean VAF**	• The average of all VAF values across all variants (e.g., for a patient with 5 variants detected, the average of the 5 VAF values from the 5 variants).<br>• Reported as summary-level statistics for all patients within a cancer type and stage category:<br>o Median<br>o Mean (SD)<br>o IQR<br>o Min, Max
**Median VAF**	• The median of all VAF values across all variants (e.g., for a patient with 5 variants detected, the median of the 5 VAF values from the 5 variants).<br>• Reported as summary-level statistics for all patients within a cancer type and stage category:<br>o Median<br>o Mean (SD)<br>o IQR<br>o Min, Max
**Max VAF**	• The maximum VAF value across all variants (e.g., for a patient with 5 variants detected, the maximum VAF of the 5 VAF values from the 5 variants).<br>• Reported as summary-level statistics for all patients within a cancer type and stage category:<br>o Median<br>o Mean (SD)<br>o IQR<br>o Min, Max
**Mean Mutant Tumor Molecules per mL**	• The mean of ctDNA molecules across all variants detected per mL of the patient's plasma<br>• Calculated as the Mean allele frequency * (extracted ctDNA in ng/volume of plasma in mL) * 330ng/genome<br>• Reported as summary-level statistics for all patients within a cancer type and stage category:<br>o Median

	○ Mean (SD)<br>○ IQR<br>○ Min, Max
**Frequency of ctDNA Detection**	• The percent of samples in which ctDNA was detected by the assay (dichotomous for yes/no detected by the assay)
**Non-Detectable Samples**	• A sample which was successfully tested, but no tumor-derived variants or alterations were detected.
